# Resource description framework technologies in chemistry

**DOI:** 10.1186/1758-2946-3-15

**Published:** 2011-05-13

**Authors:** Egon L Willighagen, Martin P Brändle

**Affiliations:** 1 Division of Molecular Toxicology, Institute of Environmental Medicine, Karolinska Institutet, SE-17177 Stockholm, Sweden; 2 Chemistry Biology Pharmacy Information Center, ETH Zürich, Wolfgang-Pauli-Str. 10, 8093 Zürich, Switzerland

## Editorial

The Resource Description Framework (RDF) is providing the life sciences with new standards around data and knowledge management. The uptake in the life sciences is significantly higher than the uptake of the eXtensible Markup Language (XML) and even relational databases, as was recently shown by Splendiani et al. [1] Chemistry is adopting these methods too. For example, Murray-Rust and co-workers used RDF already in 2004 to distribute news items where chemical structures were embedded using RDF Site Summary 1.0 [2]. Frey implemented a system which would now be referred to as an electronic lab notebook (ELN) [3]. The use of the SPARQL query language goes back to 2007 where it was used in a system to annotate crystal structures [4].

**Figure 1:**
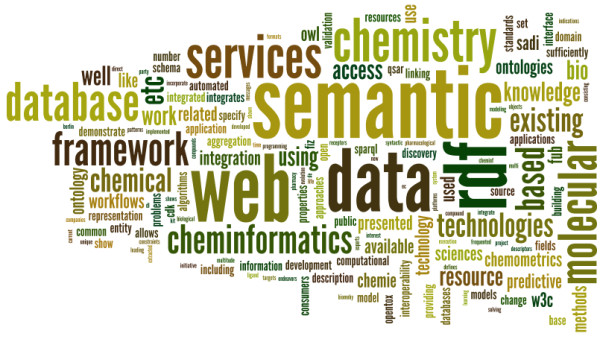
**Keyword cloud for the RDF and Computation session**.

**Figure 2:**
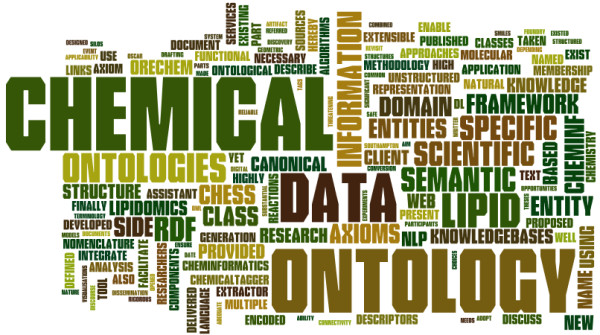
**Keyword cloud for the RDF and Ontologies session**.

**Figure 3:**
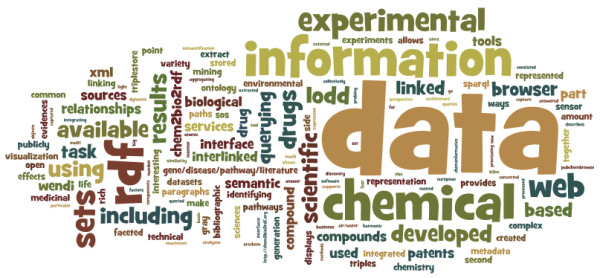
**Keyword cloud for the RDF and Chemical Applications session**.

The American Chemical Society (ACS) Division of Chemical Information (CINF) invited scientists from around the world to present their use of RDF technologies in chemistry on 22nd-23rd August 2010 at the 240th ACS National Meeting in Boston, USA. During three half-day sessions, the speakers demonstrated a mix of smaller and larger initiatives where RDF and related technologies are used in cheminformatics and bioinformatics as Open Standards for data exchange, common languages (ontologies), and problem solving. The fifteen presentations were grouped in the themes computation, ontologies, and chemical applications. Figures 1, 2 and 3 display the most important keywords reflecting the abstracts of the talks in each session as word clouds [5].

The goal of the meeting was to make more chemists aware of what the RDF Open Standard has to offer to chemistry. We are delighted to continue this effort with this Thematic Series, for which the speakers (and others) were invited to present their work in more detail to a wider chemistry community. The choice of an Open Access journal follows this goal. At this place, we would like to thank Pfizer, Inc., who had partially funded the article processing charges for this Thematic Series. Pfizer, Inc. has had no input into the content of the publication or the articles themselves. All articles in the series were independently prepared by the authors and were subjected to the journal's standard peer review process.

In the remainder of this editorial, we will briefly outline the various RDF technologies and how they have been used in chemistry so far.

## 1 Concepts

The core RDF specification was introduced by the World Wide Web Consortium (W3C) in 1999 [6] and defines the foundation of the RDF technologies. It has evolved into a set of recommendations by the W3C published in 2004 (See Table 1). RDF specifies a very simple data structure linking a subject to an object or a value (literal) using a predicate. Cheminformaticians will recognize this data structure as an edge from graph theory. This structure allows us to represent facts like "vanillin dissolves in methyl alcohol" [7]. RDF uses Uniform Resource Identifiers (URIs) to identify things. Therefore, the RDF equivalent of the solution statement could be like this so-called triple: 

http://dbpedia.org/resource/Vanillin http://example.com/dissolvesIn http://dbpedia.org/resource/Methanol.

**Table 1:** Key W3C Specifications

Year	Technology	Description
1999	RDF	Resource Description Framework (RDF) Model and Syntax Specification [6]
2004	RDF/XML	RDF/XML Syntax Specification (Revised) [13]
	RDF	Resource Description Framework (RDF): Concepts and Abstract Syntax [32]
	OWL	OWL Web Ontology Language Overview [33]
2007	OWL2	OWL 2 Web Ontology Language Document Overview [33]
2008	RDFa	RDFa in XHTML: Syntax and Processing [16]
	SPARQL	SPARQL Query Language for RDF [19]

Several of the key specifications and when they were recommended by the World Wide Web Consortium.

Since URIs may be used to reference resources on any server worldwide, RDF triples allow to span a global graph data structure. This is not surprising, since RDF is the core technology behind the proposed Semantic Web [8]. In fact, the Web nature is clear here, as one can follow both the URIs for vanillin and methanol to obtain further information on those two chemicals. These molecules' URIs are said to be dereferencable, allowing agents to spider the Web for information following the hyperlinks, quite like how you follow hyperlinks on websites. Hence, the term Semantic Web.

Recent projects such as Bio2RDF [9], Chem2Bio2RDF [10], and OpenTox [11] have brought genomic, chemical and pharmaceutical knowledge to the Semantic Web by expressing it in RDF. These three projects aim at making databases with chemical knowledge available from a central access point, interlinking the individual data sets. Smaller data sets are also becoming available as RDF, such as the Open Notebook Science Solubility data [12].

## 2 Formats

The actual use of RDF depends on various further standards. For example, standards were required that describe how RDF statements are exchanged. Several standards serve this purpose: RDF/XML is an XML-based serialization [13], while simpler formats exist with N-Triples [14] and Notation3 [15]. For integration with current web practices, RDFa has been defined to allow RDF triples to be embedded in HTML pages [16]. Additionally, a proposal has been written that describes how RDF can be serialized as Javascript Object Notation (JSON) [17], and while this is not a formal specification yet, a new RDF working group will formalize this into a new standard [18]. Several of these serialization standards are used in the papers in this Series.

Using these serializations, RDF can be downloaded directly from pure RDF documents (RDF/XML, Notation3), or extracted from RDFa-based web pages using online RDF extraction web services, like http://www.w3.org/2007/08/pyRdfa/. These approaches make it simple to aggregate chemical data from web pages.

## 3 Querying the World Wide Web

The most promising technology in the RDF family is the SPARQL Protocol and RDF Query Language (SPARQL) [19], which has been applied by Chen et al. in three chemogenomics use cases [10]. One of the use cases shows how SPARQL queries are used to find compounds that are active in bioassay for genes related to proteins to which the chemical dexamethasone binds, using information from PubChem, Uniprot, and DrugBank, all made available as RDF in the Chem2Bio2RDF database. The other use cases in this paper use the same approach by aggregating data sources before querying them. As such, it is similar to querying data stored in a relational database. However, an important difference between SPARQL and SQL query engines is the underlying data they act on: a graph of triples for RDF data, and rectangular tables in relational databases. This difference implies that RDF resources must have at least some common elements, whereas a relational DBMS assumes an identical data structure for all records of a table.

**Figure 4:**
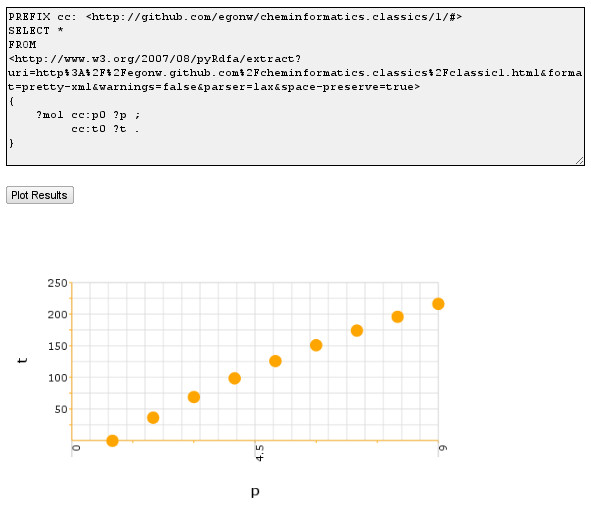
**Web page using SPARQL to visualize alkane boiling points extracted from another web page**. Web page with JavaScript by Jankowski visualizing the boiling point of a series of alkanes from Wiener [31] extracted with SPARQL from a second, XHTML+RDFa web page at http://egonw.github.com/cheminformatics.classics/classic1.html

For example, Jankowski used a public SPARQL service to extract boiling points of a series of alkanes from an XHTML webpage with the data made machine readable with RDFa, and visualized that using Javascript in another web page dynamically [20] (see Figure 4). A second important difference is that SPARQL queries can be *federated *[21]. Federated SPARQL allows one to query various RDF providers in one query. This has been used recently in the Receptor Explorer tool to help translational research by connecting basic neuroscience research with clinical trials [22]. Being able to query resources in this manner, brings us a step closer to systems biology approaches.

## 4 Ontologies

With RDF we have a data structure to link resources and provide details about those resources, and SPARQL provides us with the tools to query and aggregate that data. The next standard we will discuss now is the Web Ontology Language (OWL) which brought the RDF technology to the ontology community [23]. Ontologies are most certainly not new to chemistry [24] nor biology or life sciences, but the OWL standard makes it much easier to use ontologies, partly because they are formulated in RDF themselves. Ontologies, like controlled vocabularies and thesauri, describe what things mean, by linking terms to a human-readable definition. As such, ontologies are used for sharing knowledge in a common language, as well as to organize that knowledge. While linking resources is not new either, expressing the content of resources in explicit terms allows humans and software to reason formally on the content and to find possible sources of error. For example, Konyk et al. have used OWL to link PubChem, DrugBank, and DBPedia, noting that it offers new ways to discover knowledge [25].

There are currently not many ontologies in chemistry, but many OBO Foundry-based ontologies can be reused using an OBO to OWL mapping [26]. This makes available chemical ontologies like the CO ontology [27], the ontology of Chemical Entities of Biological Interest (ChEBI) [28,29], and the Chemical Information Ontology http://code.google.com/p/semanticchemistry/, but also other ontologies in the life sciences, such as the Gene Ontology [30]. This way, OWL provides a universal standard to link data sources in life sciences, transcending traditional boundaries between the various domains.

The current state is that different RDF resources are using different ontologies. This does not necessarily have to be a problem, because the ontologies can be explicitly mapped to each other. This way, equivalent terms from two ontologies can be formally defined as equivalent, using the OWL predicates owl:equivalentClass and owl:equivalentProperty for classes, and owl:sameAs for instance. Making the equivalence explicit this way helps to illustrate the provenance of data integration efforts.

## 5 Discussion

This Thematic Series shows the current state of the use of RDF in chemistry, as presented at the ACS RDF 2010 meeting in Boston, and provides an insight into the progress of these methods. Much of the research is currently explorative, rather than formative, though standards are being proposed. It may very well turn out that some aspects of chemistry will never be expressed in RDF, and some computation will be done without ontology-based reasoning. It is important to realize here where RDF is positioned, namely for linking resources.

However, the use of RDF for already well-defined data structures in chemistry is not obvious. Data types like connection tables and various matrices are possible, but the use of URIs makes such structures needlessly verbose. Moreover, there is no need to format already well-formalized data structures into RDF, such as the various uses of matrices in computational chemistry as RDF triples. In fact, several papers in this series outline how to combine knowledge expressed with RDF with computational services. This shows that RDF is not an isolated framework, but one that can be integrated into existing cheminformatics workflows.

What RDF does not solve, are the following issues that remain in cheminformatics. RDF is about knowledge representation, and while ontologies take care of meaning and provide requirements to verify formal data consistency, it does not enforce any data quality, data structure, or data availability. This is, in fact, similar to other ways of providing data. For example, a data set with boiling points may or may not include information about experimental error. Metabolomics data may name the molecules for which concentration profiles have been measured, or the original accurate masses from which the identity was deduced.

It must be clear, therefore, that the RDF technologies are not the solution to everything. Their use does not guarantee an impressive scientific scenario. Instead, it can help simplify data analysis and particularly data integration, making it easier to handle large volumes of data accurately, or at least, with an explicitly defined accuracy.

As such, the use of explicit, semantic formats can be considered a gold standard of scientific practise. It is about adding as much detail to your lab notebook as you need. But, it does not inhibit you from writing nonsense in your notebook.

## 6 Outlook

The future of the use of RDF technologies as open standards in chemistry looks bright, and fills the needs in chemistry for semantically linking chemical data to other data sources. RDF technologies provide a domain-independent way for representing knowledge and their open nature assures many alternative approaches for making data available as RDF. This Thematic Series shows a few novel and creative applications of these RDF technologies, and we hope they may serve as seminal work in cheminformatics for future years.
